# Comparative Sensitivity of MRI Indices for Myelin Assessment in Spinal Cord Regions

**DOI:** 10.3390/tomography11010008

**Published:** 2025-01-14

**Authors:** Philip Kyeremeh Jnr Oppong, Hiroyuki Hamaguchi, Maho Kitagawa, Nina Patzke, Kevin C. Wakeman, Khin Khin Tha

**Affiliations:** 1 Laboratory for Biomarker Imaging Science, Graduate School of Biomedical Science and Engineering, Hokkaido University, N15 W7, Kita-ku, Sapporo 060-8638, Japan; 2 Department of Radiological Technology, Hokkaido University Hospital, N14 W5, Kita-ku, Sapporo 060-8648, Japan; 3 Department of Biological Sciences, Faculty of Science, Hokkaido University, N10 W8, Kita-ku, Sapporo 060-0810, Japan; 4 Faculty of Medicine, Institute for Mind, Brain and Behavior, HMU Health and Medical University, Olympischer Weg 1, 14471 Potsdam, Germany; 5 Institute for the Advancement of Higher Education, Hokkaido University, Sapporo 060-0817, Japan; 6 Global Center for Biomedical Science and Engineering, Faculty of Medicine, Hokkaido University, N15 W7, Kita-ku, Sapporo 060-8638, Japan

**Keywords:** sensitivity, MRI, spinal cord, myelin, myelin water fraction, magnetization transfer, fractional anisotropy, mean diffusivity, electrical conductivity, relaxation time

## Abstract

**Background/Objectives:** Although multiple magnetic resonance imaging (MRI) indices are known to be sensitive to the noninvasive assessment of myelin integrity, their relative sensitivities have not been directly compared. This study aimed to identify the most sensitive MRI index for characterizing myelin composition in the spinal cord’s gray matter (GM) and white matter (WM). **Methods:** MRI was performed on a deer’s ex vivo cervical spinal cord. Quantitative indices known to be sensitive to myelin, including the myelin water fraction (MWF), magnetization transfer ratio (MTR), the signal ratio between T1- and T2-weighted images (T1W/T2W), fractional anisotropy (FA), mean diffusivity (MD), electrical conductivity (σ), and T1, T2, and T1ρ relaxation times were calculated. Their mean values were compared using repeated measures analysis of variance (ANOVA) and post hoc Bonferroni tests or Friedman and post hoc Wilcoxon tests to identify differences across GM and WM columns possessing distinct myelin distributions, as revealed by histological analysis. Relationships among the indices were examined using Spearman’s rank-order correlation analysis. Corrected *p* < 0.05 was considered statistically significant. **Results:** All indices except σ differed significantly between GM and all WM columns. Two of the three WM columns had significantly different MWF, FA, MD, and T2, whereas one WM column had significantly different MTR, σ, T1, and T1ρ from the others. A significant moderate to very strong correlation was observed among most indices. **Conclusions:** The sensitivity of MRI indices in distinguishing spinal cord regions varied. A strategic combination of two or more indices may allow the accurate differentiation of spinal cord regions.

## 1. Introduction

The spinal cord is a long, slender neural structure encased within the vertebral column, extending from the medulla oblongata at the foramen magnum of the occipital bone to the level of the second lumbar vertebra. Along its length, it is organized into cervical, thoracic, lumbar, sacral, and coccygeal regions [1]. In the transverse section, the spinal cord displays two distinct areas: central gray matter (GM), composed primarily of neuronal cell bodies, neuropils, glial cells, synapses, and capillaries, surrounded by three distinct white matter (WM) columns, which are longitudinally oriented axons organized into anterior (AC), lateral (LC), and posterior (PC) columns. Functionally, the spinal cord is an essential communication conduit, transmitting motor signals from the brain to the peripheral body, relaying sensory information from the periphery to the brain, and coordinating autonomic functions [2].

The rapid and efficient transmission of motor and sensory signals along the spinal cord is made possible by myelin, which is a protective sheath that encases axons [3,4]. Myelin’s composition—primarily lipids (about 70–80%), including cholesterol, phospholipids, and glycolipids, along with proteins (20–30%) such as the myelin basic protein (MBP) and proteolipid protein (PLP)—is crucial for its function. The lipid content raises electrical resistance and enhances signal speed, while the proteins provide structural integrity, maintaining the compact arrangement of myelin layers. Damage or abnormalities in myelin impair signal transmission, leading to symptoms such as muscle weakness, sensory loss, and coordination difficulties, which are characteristics of demyelinating diseases such as multiple sclerosis (MS) [5].

Determining myelin composition is essential for diagnosing and monitoring demyelinating diseases and other conditions that impair myelin. Traditionally, invasive histological methods such as Luxol Fast Blue (LFB) staining, osmium tetroxide (OsO_4_) staining, and transmission electron microscopy (TEM) have been considered the standard for evaluating myelin’s structure and composition [6]. While these methods provide detailed information, their invasiveness limits their clinical application, especially for ongoing monitoring. To overcome this, less invasive approaches like cerebrospinal fluid (CSF) analysis and positron emission tomography (PET) have been developed to detect myelin damage in patients [7,8]. The performance of these techniques has been satisfactory: elevated myelin basic protein (MBP) level in CSF, released during myelin degradation, serves as a promising biomarker for acute demyelination, while PET imaging using C-11-labeled N-methyl-4,4′-diaminostilbene ([^11^C]MeDAS) allows for the visualization of myelination and demyelination in vivo. However, the ultimate goal remains to develop entirely noninvasive methods that are capable of quantifying and evaluating myelin integrity reliably, enhancing diagnostic precision and patient monitoring across various clinical settings.

Advances in MRI technology, including improvements in both software and hardware, have facilitated the noninvasive assessment of myelin composition. Various MRI-based methods have been proposed, each showing potential as noninvasive markers for myelin content and demonstrating promising correlations with histological and immunohistochemical data. First, T1 and T2 relaxation times, fundamental determinants of the MRI signal, are influenced by the degree of myelination, with greater myelination typically associated with shorter T1 (longitudinal) and T2 (transverse) relaxation times [9]. This makes relaxation times useful for indirectly reflecting myelin content. Second, the ratio of T1-weighted (T1W) to T2-weighted (T2W) images can be used as a simplified metric for myelin content. T1W and T2W images generate contrasts that highlight myelin by leveraging the direct proportionality of pixel intensity to myelin in T1W images and the inverse in T2W images. This T1W/T2W ratio has been successfully investigated to evaluate the myelin content in cortical GM, assess neonatal brain myelination, quantify MS-associated tissue damage, and explore potential links to amyloid-beta accumulation [10,11,12]. Third, the myelin water fraction (MWF), obtained through an advanced 3D gradient and spin-echo sequence, analyzes T2 relaxation time to quantify the water trapped within myelin bilayers [13,14]. The short T2 relaxation time component, relative to the total T2 distribution, has been validated as an in vivo myelin biomarker, correlating strongly with histological measures. Fourth, magnetization transfer imaging (MTI), which produces a magnetization transfer ratio (MTR), enhances sensitivity to myelin by quantifying magnetization transfer between myelin macromolecules and free water [15]. MTR has significantly correlated with myelin histological measures using off-resonance radiofrequency (RF) pulses to saturate macromolecules [9]. Fifth, T1ρ, or spin-lattice relaxation in the rotating frame, is sensitive to proton exchange between the bound water in myelin and free water. Spin-locking sequences can harness this property to quantify myelin content [16].

Additional advanced MRI techniques, such as diffusion tensor imaging (DTI), provide insights into WM’s integrity through indices like fractional anisotropy (FA) and mean diffusivity (MD), reflecting water motion primarily within the extracellular spaces of myelin sheaths [17]. For high-precision spinal cord imaging, a reduced field-of-view (rFOV) DTI variant minimizes the artifacts caused by the surrounding osseous structures, enhancing spatial resolution and facilitating detailed anatomical visualization [18]. Another promising approach, electric properties tomography (EPT), uses electrical conductivity (σ) as a potential indicator of tissue integrity. Although a direct link between σ and myelin integrity is yet to be established, it is hypothesized that myelin disruption may increase ion mobility and, consequently, σ in demyelinated regions [19].

Despite the demonstrated sensitivity of these MRI techniques to myelin content and integrity, uncertainties persist in accurately characterizing regional variations in myelin composition. This study aimed to identify the most sensitive MRI index for assessing myelin composition within deer’s cervical spinal cord GM and WM. Knowledge of this index is expected to have important implications for the accurate, noninvasive evaluation of myelin and related pathologies.

## 2. Materials and Methods

### 2.1. Specimen and Preparation for MRI

**Figure 1:**
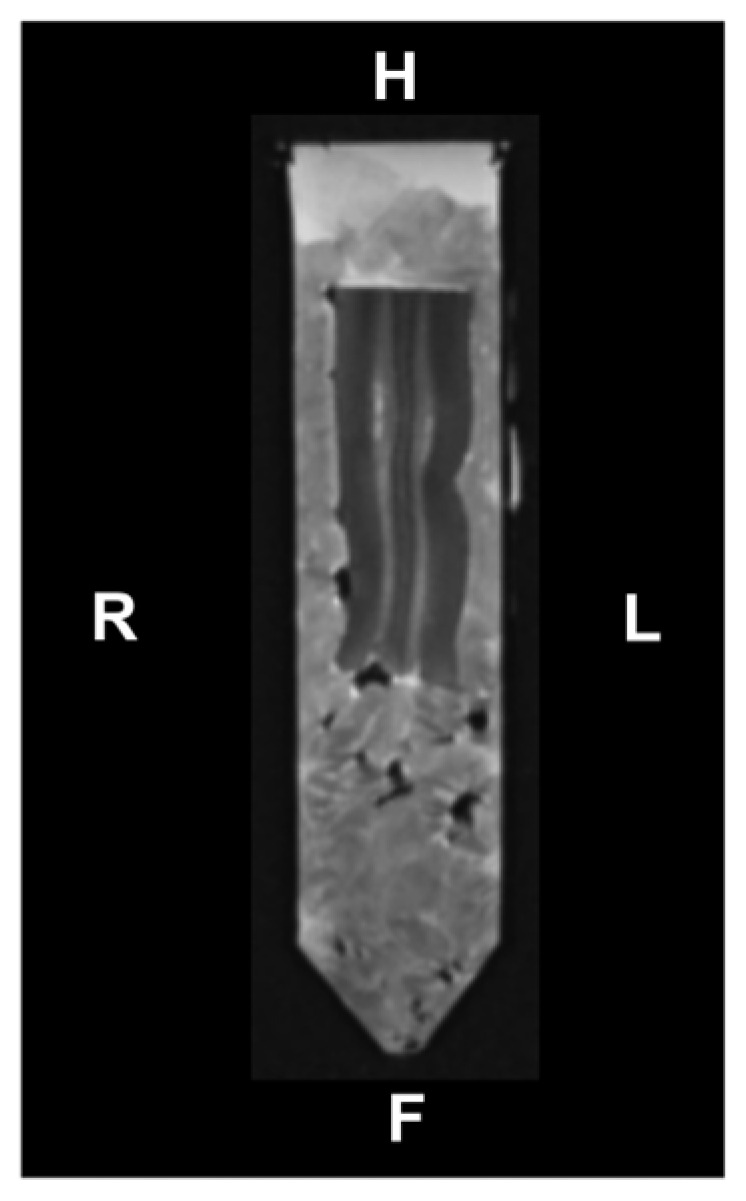
Set up of the specimen for MRI. Abbreviations: R, right; L, left; H, cranial; F, caudal.

The head and neck of a 2–3-year-old Ezo Sika Deer (Cervus nippon yesoensis) was obtained from the veterinary medicine department of the Rakuno Gakuen University within 4 h post euthanasia. The specimen was processed within 4 h post euthanasia to minimize tissue degradation and preserve structural integrity. In particular, the specimen was initially perfused through the carotid arteries with a rinse of 1 L of 0.9% saline, followed by fixation with 3 L of 4% paraformaldehyde in a 0.1 M phosphate buffer (PB) [20]. The cervical spinal cord was then taken out and post-fixed in 4% paraformaldehyde in 0.1 M PB with sodium azide for 48 h at 4 °C. It was then transferred into an 11.60 × 2.65 cm^2^ container, with the rostral end positioned superiorly and the caudal end inferiorly. The specimen was secured centrally in the container with gauze to stabilize against movements while minimizing compressions. Anatomical landmarks were used to accurately orient the specimen, with the dorsal surface facing upwards and the ventral surface facing downwards (Figure 1).

Because the specimen was obtained from the animal sacrificed for pest control [21] and was used opportunistically (i.e., the deer was not killed for research purposes), special permission to use the tissue for research was not required.

### 2.2. MRI Acquisition

The container that housed the spinal cord was placed in the MRI scanner and allowed to thaw to 22 °C. Attention was paid to match the specimen’s orientation within the scanner. Straps and pads were used to secure the container against movements during scanning. Imaging was conducted using two 3T scanners (Achieva TX, Philips Healthcare, Best, The Netherlands, and Magnetom Prisma, Siemens Healthineers, Erlangen, Germany), each equipped with a standard 32ch or 64ch head coil.

The MRI sequences comprised an axial 3D gradient and spin echo (3D-GRASE), sagittal 3D gradient echo magnetization transfer imaging (3D-GRE MTI), a sagittal magnetization-prepared rapid gradient echo (MPRAGE), axial T2-sampling perfection with application-optimized contrast (T2-SPACE), axial rFOV DTI, sagittal 3D steady-state free precession electric properties tomography (3D-SSFP EPT), sagittal magnetization prepared 2 rapid gradient echoes (MP2RAGE), sagittal Carr–Purcell–Meiboom–Gill (CPMG), and axial 3D ultrafast gradient echo T1ρ imaging (3D-GRE T1ρ). The detailed scan parameters are given in Table 1.

### 2.3. Image Processing

Following MRI scans, maps of nine quantitative MRI indices were reconstructed at the operator console or standalone workstation.

MWF maps were reconstructed voxel-wise from 3D-GRASE image data based on the MRI signal model characterized by time-dependent multi-exponential decay. This model considers distinct WM water pools, i.e., myelin and axonal (intra and extra) pools [22]. The measured signal at echo time (TE) is represented as S_TE_ = A_M_e^−TE/T2_M_^ + (A_I_e^−TE/T2_I_^ + A_E_e^−TE/T2E^), where A_M_, A_I_, and A_E_ are signal amplitudes corresponding to myelin water, intra-axonal, and extra-axonal water, respectively, and T2_M_, T2_I_, and T2_2_ are the associated transverse relaxation times. MWF maps were, thus, generated as the ratio of the myelin water signal amplitude to the total water signal amplitude: MWF = A_M_/{A_M_ + (A_I_ + A_E_)}. The reconstruction was performed using MatLab version R2014b at a standalone workstation.

MTR maps were reconstructed voxel-by-voxel from 3D-GRE MTI data based on magnetization transfer between tissue macromolecular protons and free water protons, achieved by the selective saturation of macromolecular protons with an off-resonance RF pulse. The saturation reduces the longitudinal magnetization of free water protons through continuous exchange with the saturated macromolecular pool, leading to decreased signal intensity. MTR was then calculated using the equation MTR = (M_0_ − M_sat_)/M_0_, where M_sat_ is the signal intensity after RF pulse saturation, and M_0_ is the baseline signal intensity without RF pulse saturation [23]. The reconstruction was conducted at the operator console.

T1W/T2W maps were reconstructed as the quotient of the signal intensities obtained from MPRAGE and T2-SPACE images, mathematically expressed as T1W/T2W = signal intensity from the T1W image/signal intensity from the T2W image [9,10,11,12]. Reconstruction was performed using Dr. ViewPro version 5.0 (AJS, Tokyo, Japan) on a dedicated workstation.

From rFOV-DTI data, FA and MD maps were calculated using similar principles applied in standard DTI. FA accounts for the degrees of water diffusion anisotropy within a voxel, calculated using the following formula [17]:

$$\mathrm{FA}=\sqrt{\frac{3}{2}}\frac{\sqrt{(\lambda_{1}-\mathrm{MD}{)}^{2}+(\lambda_{2}-\mathrm{MD}{)}^{2}+(\lambda_{3}-\mathrm{MD}{)}^{2}}}{\sqrt{\left({\lambda_{1}}^{2}+{\lambda_{2}}^{2}+{\lambda_{3}}^{2}\right)}}$$

The eigenvalues λ_1_, λ_2_, and λ_3_ express the diffusion tensor’s diffusivities along the three orthogonal axes, with their mean reconstructed as mean diffusivity (MD), using the equation MD = (λ_1_ + λ_2_ + λ_3_)/3. The reconstruction of both maps was performed at the main operator console of the MRI scanner.

The σ maps were reconstructed from phase images obtained using a 3D-SSFP sequence, utilizing the truncated Helmholtz equation σ = (∂x^2^ + ∂y^2^ + ∂z^2^)φ/2μ_0_ω. Here, φ represents the transceive phase, μ_0_ is the magnetic vacuum permeability, and ω is the Lamor frequency (128 MHz for 3T). Applying partial derivatives along the x, y, and z coordinates facilitates volumetric imaging, enabling EPT on a 3D-SSFP [19,24]. σ maps were calculated voxel-wise using an in-house program on a standalone workstation.

T1 maps were reconstructed from MP2RAGE imaging data using a signal model that describes the measured signal intensity at a given inversion time (TI) as S_TI_ = S_0_ (1 − 2e^−TI/T1^). S_0_ represents the equilibrium magnetization signal, and T1 is the longitudinal relaxation time [25]. Reconstruction was performed at the operator console.

T2 maps were generated from CPMG imaging sequence data using an MRI signal model described by a mono-exponential decay function of the transverse relaxation time, T2. The signal intensity at a given TE was given as S_TE_ = S_0_e^−TE/T2^, where S_0_ describes the equilibrium signal intensity, and T2 is the transverse relaxation time. The kernel of the equation given by e^−TE/T2^ describes the magnitude of signal decay after TE [26]. T2 maps were also reconstructed at the operator console.

T1ρ maps were reconstructed voxel-by-voxel from 3D-GRE T1ρ imaging data by fitting the signal across the field of view to a mono-exponential decay model as follows: S_TSL_ = S_0_e^−TSL/T1ρ^, where S_TSL_ is the magnetization signal following T1ρ spin-lock preparation for a given spin-lock duration (TSL), S_0_ represents the equilibrium magnetization signal, and T1ρ is the T1ρ relaxation time [27]. The reconstruction was performed using Jim8 software (Xinapse Systems Ltd., Essex, UK) on a standalone workstation.

Following the reconstruction of maps, those maps generated in sagittal sections, MTR, T1W/T2W, σ, T1, and T2, were resliced into 1 mm thick axial images to match other maps. This reconstruction was performed using Dr. ViewPro version 5.0.

Subsequently, the semiautomated image co-registration of all maps was performed using the default parameters of SPM12 (Wellcome Trust Centre for Neuroimaging, University College of London, Oxford, UK), running on MatLab. The maps were then checked for misregistration, and if any, they were manually corrected.

### 2.4. Measurement of Mean Values

**Figure 2:**
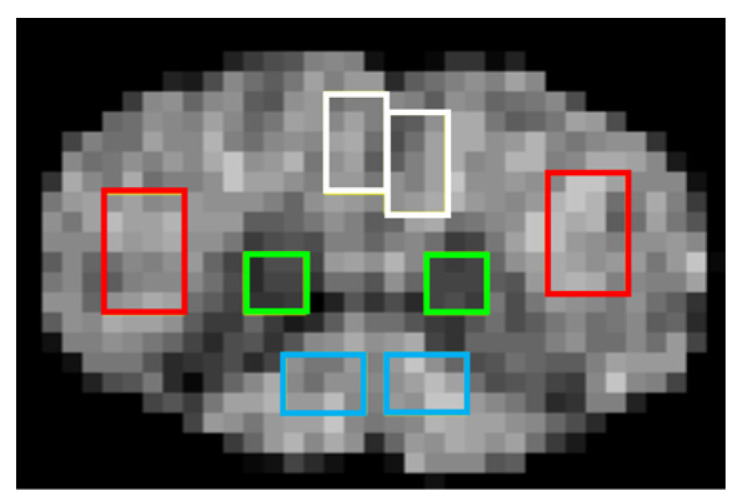
The regions of interest (ROIs) set up. ROIs for the anterior column (AC), lateral column (LC), posterior column (PC), and central gray matter (GM) are shown in white, red, blue, and green, respectively.

Rectangular regions of interest (ROIs) measuring 3.75 mm^2^, 6 mm^2^, 3 mm^2^, and 2.25 mm^2^ were placed on either side at AC, LC, PC, and GM across eight consecutive slices (Figure 2). ROIs were drawn directly on the T1W/T2W maps and allowed to superimpose onto the other coregistered maps. The ROI sizes were chosen to capture myelin distribution while ensuring robust measurements. Care was taken not to include artifacts and partial volume effects. The mean values were then extracted. Image J software (version 1.47, Wayne Rasband and contributors, National Institutes of Health, Bethesda, MD, USA) was used for this purpose.

### 2.5. Specimen Preparation for Histological Examination

Following MRI, the spinal cord was transferred to a solution of 30% sucrose in 0.1 M PB at 4 °C until it had equilibrated and was further transferred to an antifreeze solution containing 30% glycerol, 30% ethylene glycol, 30% distilled water, and 10% 0.244 M PB. Once again, the spinal cord was allowed to equilibrate in the solution at 4 °C and was then moved to a −20 °C freezer for storage until sectioning. A 3 cm block was then dissected from the spinal cord, allowed to equilibrate into 30% sucrose in a 0.1 M PB, and then frozen in crushed dry ice. The tissue block was mounted onto a metal stage and sectioned into 50 μm thick transversal sections on a sliding microtome.

The 50 μm thick specimen was then re-fixated in 4% glutaraldehyde in 0.1 M PB for 48 h at 4 °C. After re-fixation, it was equilibrated in 30% sucrose in 0.1 M PB. Following equilibration, it was transferred to an antifreeze solution containing 30% glycerol, 30% ethylene glycol, 30% distilled water, and 10% 0.244 M PB. The specimen was equilibrated again at 4 °C before being moved to a −20 °C freezer.

### 2.6. Staining

For staining, the specimen was incubated in a 1% OsO_4_ solution in 0.1 M PB for 1–2 h at room temperature. It was then thoroughly rinsed in distilled water to remove excess OsO_4_. It was then prepared for imaging by serial dehydration, resin embedding, and sectioning. The details of dehydration, embedding, sectioning, and imaging can be found in Odle et al. [28].

### 2.7. Histological Analysis

GM and WM columns of the tissue section were identified and captured using a light microscope equipped with a camera. Subsequently, high-resolution TEM images of WM columns were acquired using a Hitachi H-7650 TEM (Hitachi High-Tech Corporation, Tokyo, Japan). Two images were obtained from each ROI used in the MRI analysis. The images were then transformed into a binary system, and the myelin density of each column was measured using Image J software. Myelin density is the proportion of myelinated fibers or myelin sheaths relative to a given volume of tissue. It reflects the overall myelin content in a given region. The details about the measurement of myelin density are detailed by Huitema et al. [29].

### 2.8. Statistical Analysis

The sensitivity of each index in distinguishing GM and WM columns was evaluated using repeated measures analysis of variance (ANOVA) and post hoc Bonferroni tests or Friedman and post hoc Wilcoxon tests, depending on the normality of data distribution. For all comparisons, correction at *p* < 0.05 was considered statistically significant.

Additionally, correlations among the quantitative indices were examined to assess potential associations between myelin characterization using different techniques. The strength of correlations among the indices was determined using Spearman’s rank-order correlation analysis with statistical significance set at the corrected value of *p* < 0.05. All statistical analyses were conducted using Statistical Package for the Social Sciences (SPSS), version 26 (IBM, New York, NY, USA).

## 3. Results

### 3.1. Maps of Quantitative Indices

**Figure 3:**
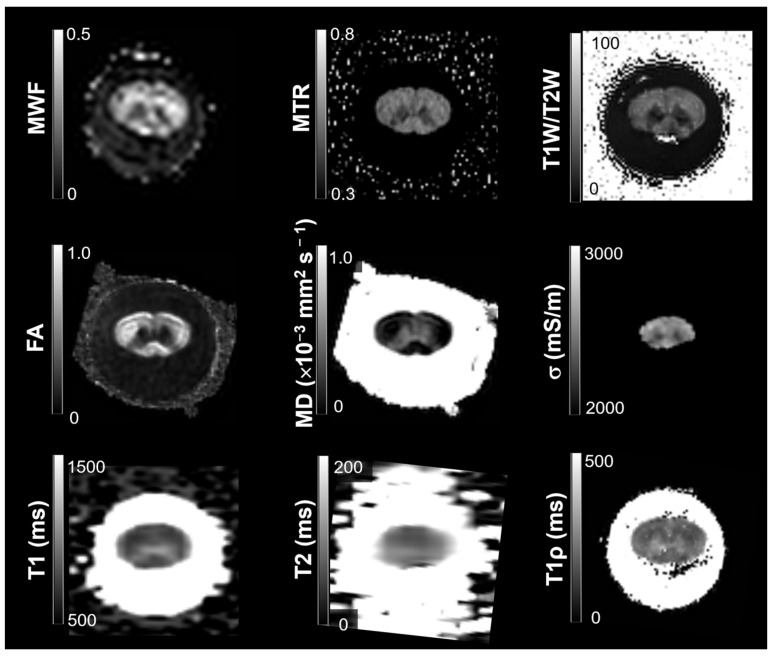
Representative co-registered maps of the quantitative MRI indices. The name of each index is given on the left side, along with the look-up table.

Representative co-registered maps of the nine quantitative MRI indices are shown in Figure 3.

### 3.2. Histological Data

**Figure 4:**
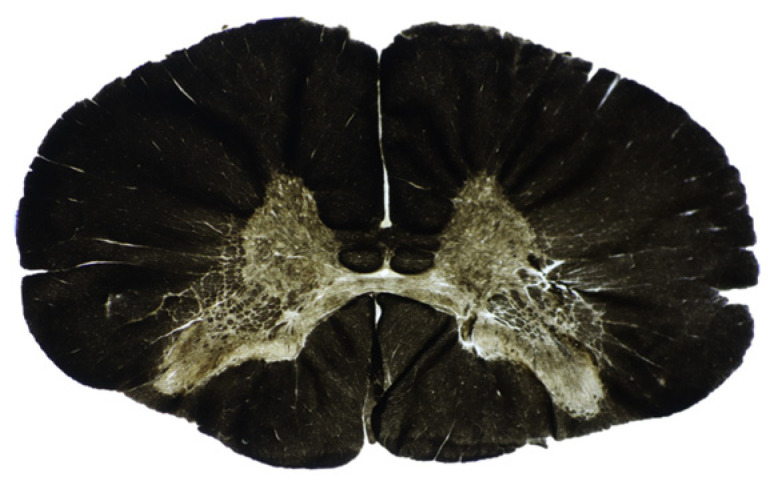
A light micrograph of the transverse section of the cervical spinal cord of deer showing clear distinction in central gray matter (GM) and white matter (WM) columns.

Figure 4 shows a light microscope image of GM and WM columns. The GM’s pale staining easily distinguished it from the WM columns. Among the WM columns, AC and LC appeared to be darker, and PC appeared to be lighter, reflecting myelin density variations.

**Figure 5:**
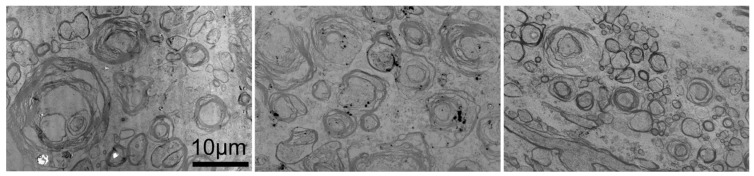
Transmission emission micrograph (TEM) of the anterior column (AC) (**left**), lateral column (LC) (**middle**), and posterior column (PC) (**right**) of the cervical spinal cord of deer.

Figure 5 shows TEM images obtained at the WM locations. The mean myelin densities and standard variation for AC, LC, and PC were 0.35 ± 0.03, 0.37 ± 0.07, and 0.32 ± 0.00, respectively.

### 3.3. The Sensitivity of Each Index in Distinguishing GM and WM Columns

**Figure 6:**
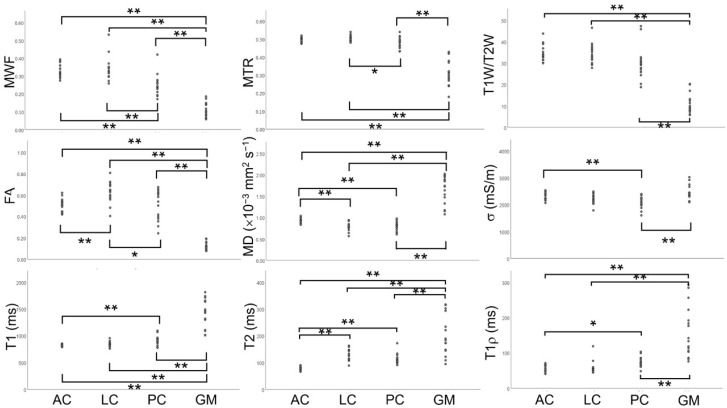
The quantitative values of MRI indices, the myelin water fraction (MWF), magnetization transfer ratio (MTR), the ratio between T1-weighted and T2-weighted images (T1W/T2W), fractional anisotropy (FA), mean diffusivity (MD), electrical conductivity (σ), and T1, T2, and T1ρ relaxation times of the central gray matter (GM) and WM columns, i.e., the anterior column (AC), lateral column (LC), and posterior column (PC). * and ** indicate corrected *p* < 0.05 and *p* < 0.01, respectively.

The mean quantitative values of MRI indices of GM and WM columns are given in Figure 6. All indices, except σ, differentiated GM from all WM columns. GM had lower MWF, MTR, T1W/T2W, FA, and higher MD, T1, T2, and T1ρ than the WM columns. The σ of GM was similar to that of AC and LC. Within the WM columns, LC had higher MWF, MTR, and FA than PC, higher FA and T2, and lower MD than AC. AC, in turn, had higher MWF, σ, and MD and lower T1, T2, and T1ρ than PC.

### 3.4. Correlations Among MRI Indices

**Figure 7:**
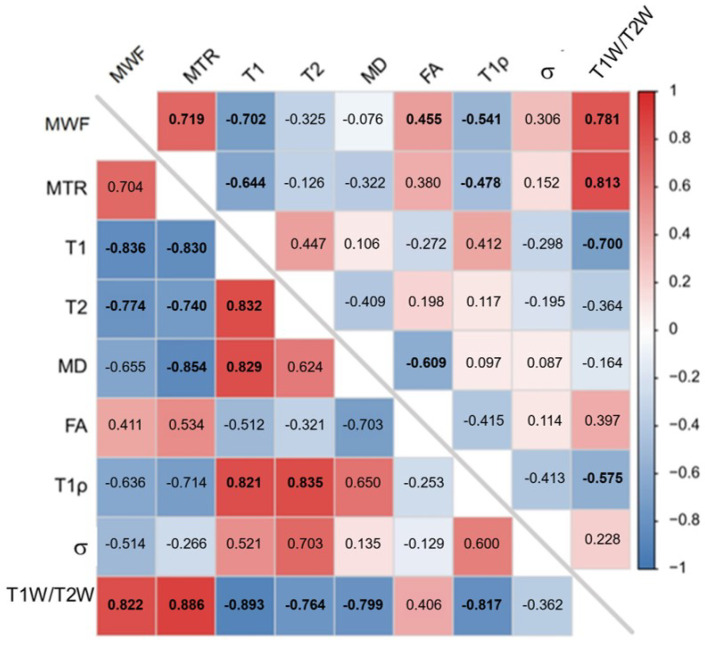
Correlation matrix showing correlation coefficients (ρ) among quantitative MRI indices. The upper triangle presents white matter (WM), and the lower triangle presents central gray matter (GM). Bolded items indicate statistical significance (corrected *p* < 0.05).

**Figure 8:**
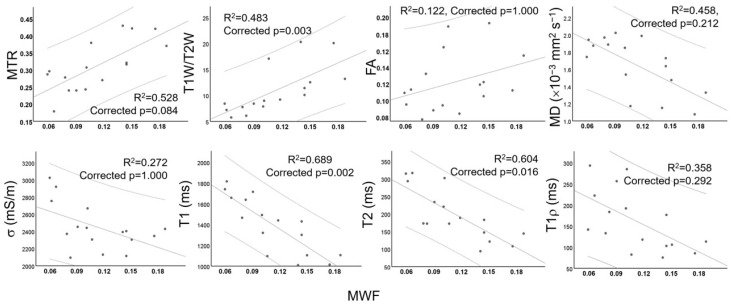
Scatterplots showing the relationships of quantitative MRI indices, the magnetization transfer ratio (MTR), the ratio between T1-weighted and T2-weighted images (T1W/T2W), fractional anisotropy (FA), mean diffusivity (MD), electrical conductivity (σ), and T1, T2, and T1ρ relaxation times of central gray matter (GM), with myelin water fraction (MWF). The mean and 95% confidence interval are given.

**Figure 9:**
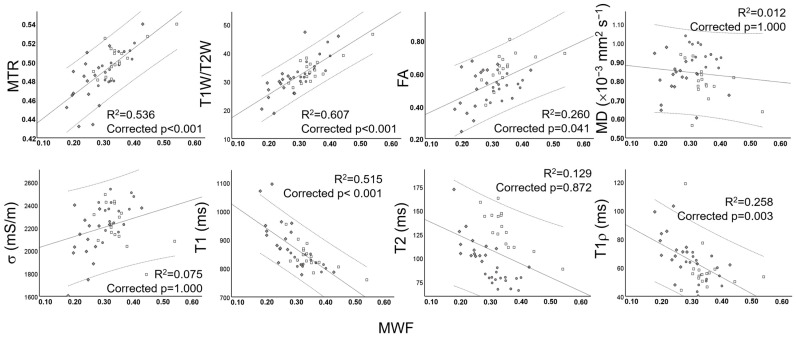
Scatterplots showing the relationships of quantitative MRI indices, the magnetization transfer ratio (MTR), the ratio between T1-weighted and T2-weighted images (T1W/T2W), fractional anisotropy (FA), mean diffusivity (MD), electrical conductivity (σ), and T1, T2, and T1ρ relaxation times of WM columns, i.e., the anterior column (AC, ●), lateral column (LC, □), and posterior column (PC, ♦), with myelin water fraction (MWF). The mean and 95% confidence interval are given.

Several pairs of significant moderate-to-strong correlations were identified, which are summarized in Figure 7. Figure 8 and Figure 9 show the relationships among quantitative MRI indices, that is, the correlation of each index with MWF, a validated myelin quantifier, within GM and WM columns.

## 4. Discussion

### 4.1. The Choice of Ex Vivo Deer Cervical Spinal Cord

This study examined the sensitivity of quantitative MRI indices in characterizing myelin within a deer cervical spinal cord. The choice of ex vivo deer cervical spinal cord tissue was guided by a few considerations, including its availability. Quadrupedal CNS tissue is often used in myelin-related studies due to its structural and functional relevance [30,31,32,33]. While interspecies differences in myelin distribution are acknowledged, this study primarily focuses on assessing the sensitivity of MRI indices to myelin distribution, which is verifiable via histological analysis. Additionally, deer spinal cord MRI offers images with high spatial resolution, minimizing partial volume effects. With its GM and WM organization closely paralleling humans, it provides translational potential. The lower compactness of myelin sheaths was observed during histological analysis, probably owing to their fixation with paraformaldehyde. Fixation with glutaraldehyde may be ideal for showing more compacted myelin sheaths, but it is believed that fixation had little effect on the total myelin density and its contribution to the corresponding MRI signal. The G-ratio is another quantitative measure that reflects myelin thickness [33], but we considered its measurement to be less suited when myelin is less compact.

Ex vivo scans, allowing a long scan time, form an indispensable part of a comprehensive comparative study like this. These scans are also free from the influence of physiological parameters such as pulsation and respiration, which is ideal for a comparison of intrinsic tissue properties. Postmortem tissue changes and microstructural changes related to the resection of the cervical spinal cord may ensue. For instance, the cessation of physiological activities may result in the decline of both T1 and T2 relaxation times [34,35]. MWF and MTR signals decrease due to diminished metabolic activity and altered macromolecule interactions [15,36]. FA and MD are affected due to slowed water diffusivities [37], and σ may decline due to disrupted ion gradients and reduced temperature. However, these changes are not focal, so such a global change in the signal would not largely impact the sensitivity of these MRI indices to identify differences in the signal among the spinal cord regions.

### 4.2. The Selection of MRI Indices

Altogether, nine quantitative MRI indices were evaluated. To our knowledge, this is the first report to compare a large number of quantitative MRI indices. The advantage of such a comprehensive comparison lies in its ability to reveal the most effective MRI indices for characterizing myelin in the spinal cord. By evaluating multiple quantitative indices, this study identified which specific indices are most sensitive to the myelin content and which provide the highest contrast and reliability. This approach not only enhances our understanding of the strengths and limitations of various indices but also aids in selecting optimal imaging parameters for future research and clinical applications involving spinal cord myelination.

### 4.3. Variable Sensitivity to Myelin Contrast

The indices demonstrated varied sensitivity in distinguishing spinal cord regions, as indicated by differences in their mean values. Nearly all indices highlighted the contrast between the GM and WM columns, reflecting their responsiveness to variations in myelin content. Across species, GM is known to contain only a few myelinated fibers, whereas WM columns are dense with myelinated axons [1]. Among the WM columns, distinct sensitivities of MWF, MTR, FA, MD, T1, T2, T1ρ, and σ in differentiating WM columns were observed. These findings suggest the diversity of myelin distribution within the WM of the deer spinal cord. The higher MWF and MTR in LC or AC and lower T1, T2, and T1ρ in AC than PC indicate higher myelin density in LC or AC. These observations agree well with myelin density measured on TEM images. The higher myelin density in LC and AC may stem from motor WM tracts running in these columns, such as the pyramidal tract [33]. WM tracts, particularly those involved in motor control, tend to have high myelination to support the fast conduction of motor signals. Similar findings with FA may stem from the same underlying factors; however, other factors, such as fiber arrangement, could also play a role [17]. The lower MD observed in the LC compared to the AC could be attributed to the larger pyramidal tract area within the LC. Additionally, the higher MD in the AC relative to the PC might be explained by differences in axonal architecture between these two regions [33]. AC often has larger, more densely packed axons aligned to support motor pathways, facilitating diffusion along the direction of these fibers. In contrast, PC primarily comprises sensory tracts with a different microstructural arrangement, potentially with more hindrance to free diffusion. This organization may lead to higher MD in AC than PC despite a higher myelin density. This may also explain the higher σ in AC since σ has been shown to have a positive correlation with MD [38]. The exact underlying mechanisms for lower T2 in AC than LC are unknown, but a difference in iron and protein concentration or glial cell population between the two may exist. The lack of a difference in T1W/T2W among the three columns may imply that this technique is not sensitive enough to characterize the tissue composition of WM [39].

### 4.4. Correlation Among MRI Indices

Several correlations were observed among the quantitative MRI indices. These correlations are thought to suggest a shared sensitivity to myelin among these indices. A lack of significant correlation between the FA and σ of GM and MD, T2, and σ of WM columns to other quantitative indices may imply that factors other than myelin density, such as the arrangement of fibers, iron or protein concentration, and glial cell population, play a role in generating tissue contrast [9,14,17].

### 4.5. Implications of This Study

Carefully selecting these quantitative MRI indices could allow for differentiating GM and all WM columns. Based on this study’s results, MWF or MTR with a DTI index (FA or MD) or T2 would suffice. DTI alone may differentiate all four regions, although factors other than myelin may also be interplayed. The choice of the MRI index to distinguish myelin composition could vary among institutions depending on the availability of MRI sequence, postprocessing tools, and the acquisition time.

This study adds several new pieces of information to the existing literature. First, very few reports have directly compared the sensitivity of various MRI indices in assessing myelin composition and validated the results with histological findings [9]. The findings of this study have valuable translational implications for human spinal cord studies, where noninvasive MRI methods are critical for assessing myelin integrity in neurological disorders, such as MS and spinal cord injuries. Second, reports of myelin distribution within the spinal cord of quadrupedal mammals, including deer, are scarce [1]. Testing these indices in a quadrupedal model like the deer is particularly insightful, as it reflects evolutionary adaptations in myelin’s structure relevant to motor and sensory function in quadrupeds, which are also functionally represented in human spinal structures. The existing literature on myelin adaptations across species has demonstrated that four-legged animals have distinct spinal column myelination patterns suited to their locomotive needs, and comparative studies can inform human models by highlighting the conserved and divergent features of spinal cord myelin composition [1,33].

### 4.6. Limitations

Several limitations need to be addressed. First, although this study successfully met its primary objective of comparing the sensitivity of various quantitative MRI indices in distinguishing spinal cord regions with varying myelin compositions, a direct correlation with actual myelin density was not accomplished. Such a correlation could have provided a stronger link between the MRI indices and myelin composition, improving the interpretation of the results. Second, the spatial resolution of a few indices may have been too low to accurately capture small or fine-grained differences in myelinated regions, potentially introducing partial volume effects that could obscure or blur distinctions between adjacent tissue types, particularly in small spinal cord columns. Third, while ex vivo samples provide a stable medium for evaluating the sensitivity of MRI indices to myelin, potential microstructural alterations in tissue necessitate the confirmation of the results by in vivo MRI scans. Lastly, these findings were based on a single deer spinal cord sample, which limits generalizability. As such, further studies with larger sample sizes and higher spatial resolution are needed to confirm the reproducibility and broader applicability of these results.

## 5. Conclusions

The sensitivity of MRI indices in assessing myelin within spinal cord regions demonstrates variability, reflecting the distinct structural and functional properties of these areas. The comparative analysis revealed that certain MRI indices such as MWF, FA, MD, and T2 are more responsive to subtle myelin changes in specific regions, while others provide sensitivity to distinguish GM and WM of the spinal cord. By strategically combining two or more indices, the assessment of myelin becomes more comprehensive, enabling more accurate differentiation among spinal cord regions. This multimodal approach may enhance our understanding of region-specific myelin integrity and contribute to improved diagnostics and treatment monitoring for spinal cord pathologies.

## Figures and Tables

**Table 1:** MRI sequences and corresponding scan parameters.

MRI Sequence	Section	TR/TE/Flip Angle (ms/ms/°)	Voxel Size (mm^3^)	Slice Thickness (mm)	FOV (mm^2^)	Number of Slices	TA (m:s)	NEX	Other Scan Parameters
3D-GRASE	axial	500/10/90	1.25 × 1.25 × 1.25	1.25	160 × 160	96	35:40	1	32 multi-echoes, echo space = 32 ms
3D-GRE MTI	sagittal	26/2.65/12	0.5 × 0.5 × 0.5	0.5	96 × 128	120	29:57	3	MT pulse on and off
MPRAGE	sagittal	1900/2.42/9	0.5 × 0.5 × 0.5	0.5	96 × 128	120	24:18	4	TI = 900 ms
T2-SPACE	axial	4000/416/120	0.5 × 0.5 × 0.5	0.5	128 × 96	288	36:52	2	ETL = 174
rFOV DTI	axial	3000/53/90	0.47 × 0.47 × 5	5	70 × 52	40	21:09	14	b = 0, 1000 s mm^−2^, diffusion gradient directions = 6
3D-SSFP EPT	sagittal	3.6/1.79/25	1.02 × 1.02 × 1	1	179 × 179	199	10:16	3	Non-selective RF pulse
MP2RAGE	sagittal	5000/5.26/5	0.5 × 0.5 × 0.5	0.5	96 × 128	120	55:26	6	TI_1_ = 935 ms, TI_2_ = 2770 ms
CPMG	sagittal	3500/13.8/180	0.78 × 0.78 × 4	4	200 × 200	45	7:52	2	5 multi-echoes, echo space = 13.8 ms
3D-GRE T1ρ	axial	3.8/1.48/12	0.63 × 0.63 × 5	5	138 × 138	27	15:37	1	Spin lock time = 0, 20, 40, 80 ms

Abbreviations: GRASE, gradient and spin echo; GRE MTI, gradient echo magnetization transfer imaging; MPRAGE, magnetization-prepared rapid gradient echo; T2-SPACE, T2-sampling perfection with application-optimized contrast; rFOV DTI, reduced field-of-view diffusion tensor imaging; 3D-SSFP EPT, 3D steady-state free precession electric properties tomography; MP2RAGE, magnetization prepared 2 rapid gradient echoes; CPMG, Carr–Purcell–Meiboom–Gill; FOV, field of view; TA, acquisition time; NEX, number of excitations; MT, magnetization transfer; TI, inversion time; ETL, echo train length; RF, radiofrequency.

## Data Availability

The data are available from the corresponding author upon reasonable request.
